# Supplementary material for: Kinsenoside Suppresses DGAT1-Mediated Lipid Droplet Formation to Trigger Ferroptosis in Triple-Negative Breast Cancer
Source: Int J Mol Sci. 2025 Mar 5;26(5):2322. doi: 10.3390/ijms26052322 (PMC11900917; doi:10.3390/ijms26052322)
Supplement: Supplementary file 1 [file ijms-26-02322-s001.zip › ijms-3445582-supplementary.pdf]

Supplementary Material

Table of Contents

Supplementary tables

Table S1. Synthesized primers for qRT-PCR.

Supplementary figures

Supplementary Figure S1. The impact of different concentrations of OA on the viability of TNBC cells.

Table S1: Synthesized primers for RT-qPCR

Gene	Forward	Reverse
ACAT1	5’-CTGGGACATCCAATTGGTAAATGC-3’	5’-AATACTGGCAAGACCGTATTCTCC-3’
ACAT2	5’- CTTCAATGGTGCCTTAGCTGC-3’	5’-CACCCACACTGGCTTGTCTA-3’
DGAT1	5’-CCCCCAACAAGGACGGAGA-3’	5’-GTTCAGGATGCCACGGTAGT-3’
DGAT2	5’-CAAGAAAGGTGGCAGGAGGT-3’	5’-GGTCAGCAGGTTGTGTGTCT-3’
BSCL2	5’-TCTCGCTGACTAAGGGTGGA-3’	5’-GATTCACAGGGGACTCTGGC-3’
LDAF11	5’-ACTTGGACAGCCATCCGTTT-3’	5’-TAAGCACCACGATGAGCAGG-3’
PLIN1	5’-CTGGAGGAAAAGATCCCCGC-3’	5’-ATGGGAACGCTGATGCTGTT-3’
PLIN2	5’-CTGCTCTTCGCCTTTCGCTG-3’	5’-ACCACACTCGGTTGTGGATCA-3’
ATGL	5’-CCCACTTCAACTCCAAGGAC-3’	5’-TAGAGTGGCAGGTTGTCTGA-3’
HSL	5’-CTCCTGCACAAATCCCGCTA-3’	5’-CGTCGCCCTCAAAGAAGAGT-3’
MAGL	5’-CAACTGCTGAATGCCGTCTC-3’	5’-GGTAGGCACCTTCATAAATCTTG-3’
GAPDH	5’-GGAGCGAGATCCCTCCAAAAT -3’	5’-GGCTGTTGTCATACTTCTCATGG -3’

Supplementary Figure S1. The impact of different concentrations of OA on the viability of TNBC cells.

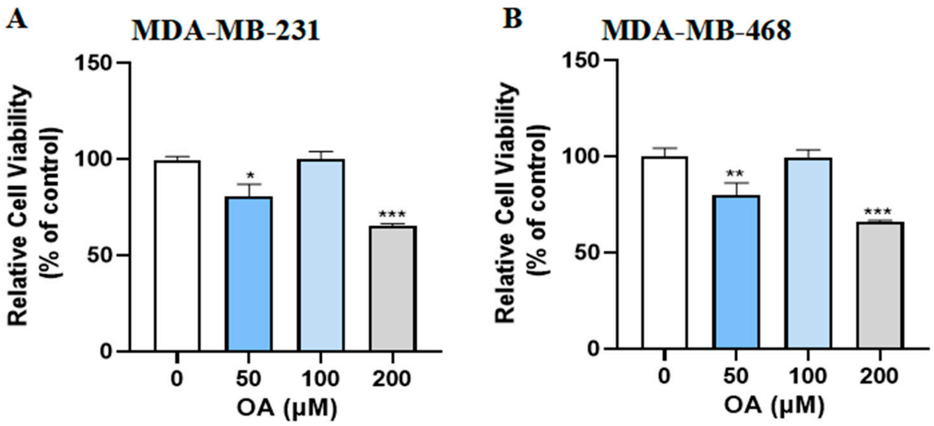

Supplementary Figure S1. The impact of different concentrations of OA on the viability of TNBC cells. (A) MDA-MB-231 and (B) MDA-MB-468 cells were treated with OA (0, 50, 100 and 200μM) for 48 hours, after which the cell viability was determined using MTT (n=3). All experimental data were presented as mean ± SD. * $p < 0.05$, ** $p < 0.01$ and *** $p < 0.001$ vs. control group.
